# Macroautophagy and Selective Mitophagy Ameliorate Chondrogenic Differentiation Potential in Adipose Stem Cells of Equine Metabolic Syndrome: New Findings in the Field of Progenitor Cells Differentiation

**DOI:** 10.1155/2016/3718468

**Published:** 2016-12-08

**Authors:** Krzysztof Marycz, Katarzyna Kornicka, Jakub Grzesiak, Agnieszka Śmieszek, Jolanta Szłapka

**Affiliations:** ^1^Electron Microscopy Laboratory, The Faculty of Biology and Animal Science, Wroclaw University of Environmental and Life Sciences, Wroclaw, Poland; ^2^Wroclaw Research Centre EIT+, Wroclaw, Poland

## Abstract

Equine metabolic syndrome (EMS) is mainly characterized by insulin resistance, obesity, and local or systemic inflammation. That unfriendly environment of adipose tissue has huge impact on stem cells population (ASC) residing within. In the present study, using molecular biology techniques and multiple imaging techniques (SEM, FIB-SEM, and confocal microscopy), we evaluated the impact of EMS on ASC viability and chondrogenic differentiation. Moreover, we visualized the mitochondrial network and dynamics in ASC_CTRL_ and ASC_EMS_ during control and chondrogenic conditions. In control conditions, ASC_EMS_ were characterized by increased mitochondrial fission in comparison to ASC_CTRL_. We found that extensive remodeling of mitochondrial network including fusion and fission occurs during early step of differentiation. Moreover, we observed mitochondria morphology deterioration in ASC_EMS_. These conditions seem to cause autophagic shift in ASC_EMS_, as we observed increased accumulation of LAMP2 and formation of multiple autophagosomes in those cells, some of which contained dysfunctional mitochondria. “Autophagic” switch may be a rescue mechanism allowing ASC_EMS_ to clear impaired by ROS proteins and mitochondria. Moreover it provides a precursors-to-macromolecules synthesis, especially during chondrogenesis. Our data indicates that autophagy in ASC_EMS_ would be crucial for the quality control mechanisms and maintenance of cellular homeostasis ASC_EMS_ allowing them to be in “stemness” status.

## 1. Introduction

Equine metabolic syndrome (EMS), the most common metabolic disorder in horses, is characterized by insulin resistance, obesity and abnormal fat deposition, chronic or past laminitis, and finally local and/or systemic inflammation. According to the current statistics, obesity in horses affects over 45% of the population and is steadily growing [1]. Increasing number of sport horses suffer from EMS due to high starch diet (rich in cereals), elevated environmental stress leading to excessive cortisol production, and free radicals that impair their self-repair antioxidative defense. In parallel to endocrine disorders, the musculoskeletal disorders (MSDs) are the most common in the field of equine veterinary regenerative medicine. Recently, mesenchymal stem cells harvested from adipose tissue are extensively investigated and considered as a most promising regenerative tool for both MSDs and endocrine treatment including EMS. Data indicate the beneficial effects of mesenchymal stem progenitor cells (MSC) based therapies in the course of diabetes type II, though still focusing on rodents model [2, 3]. It has been proven that MSC are capable of improving metabolic control, decreasing insulin requirements, ameliorating insulin sensitivity, and increasing islets numbers in the pancreas [4, 5]. In turn, many independent clinical trials—including our own—showed that in the field of MSD treatment the MSC-based therapies have positive clinical outcomes [6]. However, the effectiveness of cellular therapies might be strongly limited by the physiological condition of engrafted cells because it affects their phenotypic plasticity (multipotency), aging, senescence, and finally oxidative stress factors accumulation that have great role in pathogenesis of both MSDs and EMS disorders.

Currently, the adipose tissue has become the most popular source of mesenchymal stem cells used for cellular therapies in the wide field of regenerative veterinary medicine of both small and large animals. These cells are characterized by the presence of specific surface markers including CD90, CD105, and CD44 and lack of CD45 expression. Other features of MSCs include their elevated proliferative potential, increased viability, and high clonogenic potential (CFU-fs) in conjunction with high ability to self-renew [7, 8]. Their multipotent properties as well as self-renewal and proliferative potential are maintained by expression of the following transcripts: Oct4 (Octamer Binding Transcription Factor-4), SOX2 (SRY (Sex Determining Region Y) Box-2), and telomerase reverse transcriptase (TERT). The genes mentioned above are the most important multipotency/pluripotency regulators that control tissue homeostasis and ensure regeneration and tissue repair. Moreover, the last marker, TERT, is in particular responsible for the regulation of lifespan, allowing for indefinite division without shortening of telomeres [9–12]. The regenerative potential of ASCs is explained* inter alia *by their paracrine/autocrine activity. In particular, synthesis and secretion of extracellular microvesicles (ExMVs) that are rich in growth factors including bone morphogenetic protein (BMP-2), vascular endothelial growth factor (VEGF), and finally fibroblast growth factor (FGF) [5, 13]. All these molecules are crucial regulatory proteins, essential for regenerative potential of the organism; therefore their secretion and biological activity may be important in the context of MSD as well as EMS treatment. For example, recently, the FGF21 has been shown to improve glucose tolerance, lower serum free fatty acids, and lead to weight loss in obese mice. Thus, serum FGF level seems to be relevant mediator of fatty acid oxidation and lipid metabolism. Moreover, ExMVs also contain antiapoptotic and anti-inflammatory factors such as interleukin 10 (IL-10), transforming growth factor beta (TGF-*β*1), and interleukins 4 and 13 (IL-4, IL-13) [14–16].

The MSC may influence on adjacent cells not only by the production of the ExMVs carrying proteins and lipids, but also with ExMVs enriched in nucleic acids, most importantly mRNA and miRNA [17]. Circulating extracellular miRNA molecules are considered not only as mediators of cell-cell communication, but also as biomarkers of physiological and pathological processes. The main reason for that is the extreme evolutionary conservation of miRNA sequences and their role as regulators of important cellular processes, such as proliferation and differentiation—strongly associated with healing and regenerative potential of the organism. The additional argument is that miRNAs are selectively incorporated into the ExMVs; therefore the information concerning the miRNA level may have diagnostic potential in various diseases, including EMS. In this context miRNA are investigated as molecules regulating insulin signaling, immune-mediated inflammation, adipokine expression, adipogenesis, lipid metabolism, and food intake regulation. Recently, the expression of few miRNAs was linked with the development of type II diabetes (T2D) [9]. The miRNAs, identified as potentially important in pathogenesis of T2D, include miR-223, 489 or 140, and 146a. Their expression level has been correlated mainly with proinflammatory target genes in obese and T2D patients.

The progressive oxidative stress and inflammation that are characteristic of insulin resistance conditions induce in progenitor cells of EMS horses the natural mechanism that protects them against cellular damage and apoptosis. Recently, the autophagy has been reported as such mechanism that prevents the metabolically affected progenitor cells from extracellular and intracellular stresses and allows them to maintain their natural multipotency. Autophagy, as a dynamic process, might be divided into few stages including (i) induction of the process, (ii) autophagosome formation, (iii) autophagolysosome formation, and (iv) delivery and degradation of the autophagic body. However, it seems that the autophagy process is not always associated with autolysosome formation and does not necessarily lead to apoptosis. The hypothesis proposed is that EMS conditions might temporarily induce autophagy in stem progenitor cells, allowing them to keep physiological functions and survive [10]. In the course of the autophagy several genes and their products, called ATG and atg, respectively, are involved in its regulation. One of the central players that initiate autophagy, through interaction with class III PI3K, is Beclin1. In turn LAMP2 enhanced formation of autophagosomes and autolysosomes. The process of autophagy starts when membrane, also known as a phagophore, is isolated predominantly from lipid bilayer derived from endoplasmic reticulum (ER) and/or the trans-Golgi and endosomes [11]. Emerging data now indicate that ER stress might be a potent autophagy inducer, resulting in physiological and functional recycling of number of organelles, including mitochondria. Regarding the background, the process of autophagy may counterbalance the ER expansion resulting from cellular stress, may enhance cell survival, or commit the cell to nonapoptotic death. Another way of efficient cellular protective process is the selective autophagy of mitochondria, known as mitophagy [12]. This mechanism is an important mitochondrial quality control eliminating damaged mitochondria. Both nonselective and selective autophagy are a potential lifebuoy for progenitor cells, maintaining and keeping their proliferative and differentiation potential, especially in course of pathological conditions like EMS.

Bearing in mind postulates mentioned above, we were interested whether adipose-derived progenitor cells of EMS horses that are affected by elevated oxidative stress—of both mitochondrial and endoplasmatic reticulum—exhibit impaired multipotency and are capable of developing automechanism that protects them from molecular quiescence and allow them to maintain their functionality. Our hypothesis is based on an assumption that macroautophagy and selective mitophagy are the mechanisms maintaining cellular homeostasis of ASC derived from EMS horses during their differentiation into chondrogenic cells, thus keeping their functionality and maintaining regenerative potential. Another proposed explanation is the horizontal transfer of ExMVs containing miRNA that regulate gene expression in dysfunctional cells and promoting their multipotent recovery. Here, we demonstrated that EMS conditions impair chondrogenic differentiation potential of ASCs and finally cause extracellular matrix formation dysfunctions. Moreover, we have fund that EMS horses suffer for reduced mitochondrial biogenesis, limited fusion, and abundance of autophagosomes and autolysosomes formation that affects their “stemness.” Still, more investigations and effort are required to stop or what is more required reverse the senescence stage of ASC_EMS_ cells to use them in clinical practice.

## 2. Materials and Methods

All reagents used in this experiment were purchased from Sigma Aldrich (Poland), unless indicated otherwise.

All experimental procedures were approved by the II Local Ethics Committee of Environmental and Life Sciences University (Chelmonskiego 38C, 51-630 Wroclaw, Poland; decision number 84/2012).

### 2.1. Animals Qualifications

All horses were age-matched (mixed sex, 9–14 years; mean ± SD, 11.2 ± 1.7 years) and divided into two groups: EMS group (*n* = 6) and control, healthy horses (*n* = 6). Detailed characterization of animals used in this study is shown in Table 1. Qualification to the experimental groups was performed based on (i) extensive interviews with owners, (ii) measurement of body weight, (iii) estimation of body condition score (BCS) and cresty neck scoring system (CNS), (iv) palpation and visual assessment of the hoof capsule, (v) X-ray examination, (vi) resting insulin levels, (vii) combined glucose-insulin test (CGIT), and (viii) LEP concentration as previously described by Basinska et al. [18].

### 2.2. Isolation of Adipose-Derived Mesenchymal Stem Cells (ASC)

White, subcutaneous adipose tissue (2 grams) was collected from the horses' tail base, according to the standard surgical procedure and ethical standards, as previously described. After harvesting, specimens were placed in a sterile Hank's balanced salt solution (HBSS). Cells were isolated under aseptic conditions following the previously described protocol by Grzesiak et al. [7]. Briefly, tissue samples were first cut into small pieces and minced using surgical scissors. Then, the extracellular matrix was digested with collagenase type I (1 mg/mL) for 40 minutes at 37°C and 5% CO_2_. Homogenates were next centrifuged at 1200 ×g for 10 minutes at room temperature (IEC CL31R, Thermo Scientific). The supernatant was discarded and the pellet was resuspended in the culture medium. The cell suspension was then transferred to a culture flask.

### 2.3. Cell Culture

During the experiment, the cells were cultured under aseptic and constant conditions in an incubator (37°C, 5% CO_2_, and 95% humidity). DMEM containing 4500 mg/L glucose supplemented with 10% FBS and 1% of PSA was used as a culture medium. The media were changed every two days and the cells were passaged using trypsin solution (TrypLE™ Express; Life Technologies) after reaching 80% confluence. Prior to the experiment, EqASCs were passaged three times. All experiments were performed after 7 days of ASC propagation.

### 2.4. Immunophenotyping Using Flow Cytometry

Equine ASC were recognized by immunophenotyping using fluorochrome conjugated monoclonal antibodies specific for CD44, CD45, CD90, and CD105. Due to immunophenotyping ASC were detached using TrypLE Express solution, washed with HBSS, and resuspended at total of 5 *∗* 10^5^ cells/mL. Cell suspension was incubated at 4°C for 20 minutes with the specific antibodies preconjugated with peridinin chlorphyllprotein (PerCP) and fluorescein isothiocyanate (FITC) (anti-CD105, Aris, SM1177PT; anti-CD45, Novus Biologicals, NB1006590APC; anti-CD44, R&D Systems, MAB5449; and anti-CD90, Abcam, ab225). At least ten thousand stained cells were acquired and analysed by Becton Dickinson FACSCalibur flow cytometer. The samples were analysed using CellQuest Pro software.

### 2.5. Multipotency Assay

To confirm multilineage differentiation potential of ASC, cells were cultured in in StemXVivo kits (R&D Systems) in accordance to manufacturer's instructions. In order to perform the test, the cells were seeded in a 24-well plate at the initial density of 1 × 10^4^ and the media (500 *μ*L/per well) were changed every two days. Osteogenesis and chondrogenesis lasted 10 days, while adipogenesis lasted 9 days. Extracellular mineralized matrix was visualized with Alizarin Red while formation of proteoglycan-rich matrix was confirmed with Safranin O staining. Lipid droplets formed during adipogenesis were stained with LipidTox (Life Technologies).

### 2.6. Assessment of Cell Proliferation Rate

Cell proliferation rate was established with resazurin-resorufin test. In order to perform the assay, cells were seeded at concentration 2 × 10^4^ per well in 24-well culture plates. The proliferation rate was evaluated after the 1st, 5th, 7th, and 10th days of the experiment. First, culture medium was replaced with a medium containing 10% of resazurin (Alamar Blue). Next, cells were incubated with dye for 2 hours at 37°C. Then, supernatants were collected and transferred to 96-well plates. Dye reduction was determined using a spectrometer (BMG Labtech) at the specific wavelengths, that is, 600 nm for resazurin and 690 nm as a background absorbance. Cell number was obtained from test data. To prepare the curve, cells were seeded at the density of 2 × 10^4^, 4 × 10^4^, and 8 × 10^4^ per well and dye absorbance was measured in relation to certain cells number. Linear trendline equation allowed estimating cells number throughout the experiment. Proliferation factor (PF) of cells was determined in relation to the proliferation activity of ASC isolated from healthy individuals. The value equal to 1 is normative unit, showing proliferative activity of ASC from control group.

### 2.7. Scanning and Transmission Electron Microscopy

Detailed cells morphology was evaluated using SEM. Cells were fixed with 4% paraformaldehyde (PFA), rinsed with distilled water, and dehydrated in a graded ethanol series (concentrations from 50% to 100%, every 5 min). Samples were then sprinkled with gold (ScanCoat 6, Oxford), transferred to the microscope chamber, and observed using SE1 detector, at 10 kV of filament tension.

For transmission electron microscopy (TEM), cells were fixed in 2.5% glutaraldehyde at 4°C. After fixation cells were (1) centrifuged at 2000 ×g for 10 min and rinsed with PBS for 30 min, (2) centrifuged again, parameters as above, (3) incubated with 1% osmium tetroxide in HBSS for 2 hours, (4) washed with HBSS, (5) centrifuged as described above, (6) dehydrated in graded acetone series (30% to 100%), and (7) embedded using Agar Low Viscosity Resin Kit (Agar Scientific Ltd., Essex, UK). Ultrathin sections (80 nm) were collected on copper grids. Uranyl acetate and lead citrate were used for contrasting for 30 and 15 min, respectively. The observations were carried out using Auriga60 Zeiss STEM, at 20 kV filament tension.

### 2.8. FIB-SEM Analysis

To analyse the volume of cell interiors at the mitochondria sites, FIB-SEM analysis used modified method previously developed by Knott et al. [19]. For ultrastructural observations, cells from EMS- and non-EMS groups were cultured on 6 mm round coverslips to semiconfluence. Next, cells were fixed with 2.5% glutaraldehyde in 0.1 M cacodylate buffer (pH = 6.8) for one hour at 4°C. After fixation, cells were washed three times with 0.1 M cacodylate buffer (pH = 6.8), followed by incubation on ice in 2% osmium tetroxide/1.5% potassium ferricyanide in cacodylate buffer for one hour. Next, samples were washed three times with buffer and two times with ultrapure water, followed by their incubation in 1% uranyl acetate aqueous solution at 4°C overnight. After incubation, cells were washed three times in ultrapure water and dehydrated in graded ethanol series. Next, samples were infiltrated with resin (Agar Low Viscosity Resin Kit, Agar, UK) by subsequent change of resin-to-ethanol proportions every two hours at room temperature (1 : 3, 1 : 1, and 3 : 1), followed by incubation in pure resin for two hours. After infiltration of cells, resin was polymerized for 36 h at 60°C. Resin blocks were then detached from coverslips by plunging the samples in liquid nitrogen, followed by quick heating in hand and tapping the preparations. Resin blocks with recovered cell-containing surfaces were mounted on microscope stubs with carbon tape, maintaining the horizontal position of recovered surfaces, then sputtered with 20 nm layer of gold (Leica EM ACE600), and placed in field-emission cross beam electron/ion microscope (Zeiss Auriga 60). Prior to focused ion beam (FIB) milling, the areas containing chosen cells were covered with ~50 nm protective layer of platinum. The first coarse trench was prepared with 30 kV/16 nA aperture, while the polishing during slice and view technique was done using 30 kV/2 nA aperture. The imaging of subsequent cellular features was conducted using SE2 detector at 2 kV of electron beam voltage. Six representative images of mitochondria reflecting the 600 nm of length on* z*-axis were captured for each experimental group, giving the coarse picture of about 2400 nm × 1800 nm × 600 nm (*x*-axis  × * y*-axis  × * z*-axis) of volume.

### 2.9. Confocal Microscopy Imaging

In order to visualize the mitochondria, cells were incubated with MitoRed dye (1 : 1000) in 37°C for 30 minutes. Following fixation with PFA, cells were rinsed three times with HBSS and cells' nuclei were counterstained with diamidino-2-phenylindole (DAPI; 1 : 1000 in HBSS) for 5 minutes.

Prior to the analysis of LAMP2 localization, cells were fixed in 4% PFA for 30 min and washed three times with HBSS. Then, cells' membranes were permeabilized with 0.5% Triton X-100 for 20 min at room temperature. After washing with HBSS three times, unspecific binding sites were blocked with blocking buffer (10% Goat Serum, 0.2% Tween-20 in HBSS) for 45 min. Cells were then incubated overnight at 4°C with primary antibodies against LAMP2 (Abcam) diluted 1 : 500 in HBSS containing 1% Goat Serum and 0.2% Tween-20. Cells were then washed again and incubated for 1 hour with goat anti-mouse secondary antibodies conjugated with atto-488 (dilution 1 : 1000, Abcam), avoiding direct light. Subsequently, nuclei were counterstained by incubation with DAPI for 5 min. Cells were observed and photographed using confocal microscope (Observer Z1 Confocal Spinning Disc V.2 Zeiss with live imaging chamber) and analysed using ImageJ software.

### 2.10. Flow Cytometry Analysis

To investigate the expression of LAMP2 and Ki67, cells were detached from culture plate and centrifuged (350 ×g for 5 minutes) followed by the 10-minute fixation in 4% ice cold PFA. After washing, cells were then incubated in 0.1% Tween-20 in HBSS for 20 min. Then cells were washed again and incubated with anti LAMP2 (Abcam) and Ki67 (Abcam) antibody, diluted 1 : 200 in HBSS containing 10% Goat Serum for 30 min at 22°C. Following another wash, cells were incubated with Alexa 488 goat anti-mouse secondary antibodies (1 : 500, Alexa Fluor 488, Abcam) for 30 minutes at 22°C. At least five thousand stained cells were acquired and analysed by FACSCalibur flow cytometer. The samples were analysed using CellQuest Pro software.

To estimate the mitochondrial membrane potential, cells were detached from culture dishes and incubated with 1 mM JC1 (Life Technologies) for 30 min in 37°C. After washing, cells were analysed in FACSCalibur flow cytometer. At least five thousand stained cells were acquired and analysed using CellQuest Pro software.

### 2.11. Analysis of Extracellular FGF-21 Levels

To evaluate the serum levels of FGF-21, enzyme-linked immunosorbent assay (ELISA) was performed. In order to evaluate the amount of FGF-21, blood was harvested from investigated individuals and centrifuged prior to serum isolation. The ELISA test was purchased from MyBioSource and performed in accordance to the manufacturer's instructions.

### 2.12. NO, SOD, and ROS Analysis

Nitric oxide concentration was assessed using commercially available Griess Reagent Kit (Life Technologies). Superoxide dismutase (SOD) activity was measured using a SOD Assay kit (Sigma Aldrich). Reactive oxygen species (ROS) were estimated by incubating cells with an H2DCF-DA (Life Technologies). All procedures were performed in accordance to the manufacturer's instructions.

### 2.13. Quantitative Real-Time Reverse Transcription Polymerase Chain Reaction (qRT-PCR)

After the 7th day of culture (viability test) and the 11th day (chondrogenic differentiation), the cells were rinsed with HBSS and homogenized by TriReagent®. Total RNA was isolated using phenol-chloroform method as previously described by Chomczynski and Sacchi [20]. The obtained RNA was then diluted in DEPC-treated water and analysed in terms of amount and quality using a nanospectrometer (WPA Biowave II). Genomic DNA digestion and cDNA synthesis were performed using PrimeScript kit (Takara, Clontech). For each reaction, 150 ng of total RNA was used. Both processes were performed in accordance with the manufacturers' instructions using a T100 Thermal Cycler (Bio-Rad). The qRT-PCR reactions were performed using a CFX Connect^TM^ Real-Time PCR Detection System (Bio-Rad). Reaction mixture contained 2 *μ*L of cDNA in a total volume of 20 *μ*L using SensiFAST SYBR & Fluorescein Kit (Bioline). The concentration of primers in each reaction equaled 500 nM; primer sequences used in individual reactions are listed in Table 2. Relative gene expression analysis (Qn) was calculated in relation to the GAPDH housekeeping gene.

### 2.14. Analysis of MicroRNA (mir) Expression

Total RNA was isolated by TRI Reagent (Sigma Aldrich) extraction method. The precipitation was performed overnight at −20°C in order to increase the yield of small RNAs. Concentration and purity (A260/A280 ratio) of total RNA were measured using nanospectrometer (WPA Biowave II). Traces of genomic DNA (gDNA) were digested with DNA-free™ Kit (Ambion). To determine miRNA expression, 375 ng of RNA was reverse-transcribed using Mir-X miRNA First-Strand Synthesis Kit (Clontech Laboratories, Inc.). The matrices were used for quantitative PCR with SYBR Advantage qPCR Premix (also derived from Clontech Laboratories, Inc.). Specific miRNA primers used for detection of miR-140, miR-146a, miR-223, and miR-489 are listed in Table 3. The following cycling conditions were applied during reaction: 95°C for 10 seconds, followed by 55 cycles of 95°C for 5 s and annealing temperature 60°C for 20 s with a single fluorescence measurement. To determine the specificity of the PCR products, analysis of the dissociation curve of amplicons was performed. Melting curve was determined with a program ramped up from 55 to 95°C at a heating rate of 0.2°C/s and continuous measurement of the fluorescence. Normalization of miRs expression (Qn) was performed in relation to U6snRNA. Both RNA purification and cDNA synthesis were performed using a T100 Thermo Cycler, while qPCR was performed on CFX Connect Real-Time PCR Detection System (all platforms originated from Bio-Rad).

### 2.15. Isolation of Microvesicles (MVs)

MVs were isolated in accordance to protocol presented by Shin et al. [21], which delivers high yield of extracellular vesicles using aqueous two-phase system composed of dextran (DEX) and polyethyleneglycol (PEG). Briefly, the culture medium is phase-separated by centrifugation at 1,000 ×g for 10 min at RT. The top phase is composed of PEG-rich solution and the bottom phase is composed of dextran-rich solution (this phase contains MVs). Isolated MVs were then homogenized with TriReagent® and total RNA was isolated prior to qRT-PCR reaction.

### 2.16. Statistical Analysis

All experiments were performed in triplicate or more. Statistical analysis was performed using GraphPad Prism 5 software (La Jolla, USA). Differences between groups were determined using unpaired Student's *t*-test. Differences with a probability of *p* < 0.05 were considered significant.

## 3. Results

### 3.1. Immunophenotyping and Multipotency Assay

**Figure 1:**
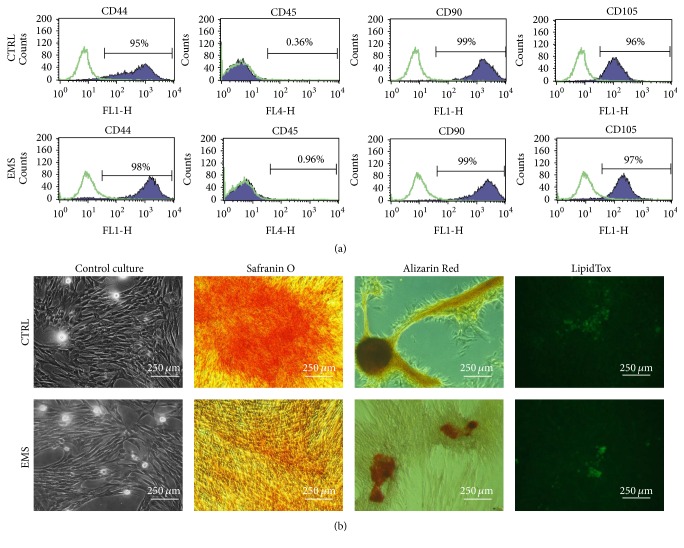
Characterization of isolated ASC analysis of surface antigens and multipotency assay. Using flow cytometer, the expression of CD44, CD45, CD90, and CD105 antigens was investigated. Isolated cells were characterized by the expression of CD44, CD90, and CD105 while they lacked the expression of CD45 hematopoietic marker (a). Interestingly, ASC_EMS_ displayed higher expression of CD44. Multipotency of ASC was confirmed by trilineage differentiation assay. Representative photographs showing the effectiveness of chondrogenesis (Safranin O), osteogenesis (Alizarin Red), and adipogenesis (LipidTox) (b). Cells cultured in standard culture medium served as a control.

Isolated cells presented typical for ASC surface antigens characteristics including expression of CD44, CD90, and CD105 and the lack of expression of CD45 surface antigen (Figure 1(a)). Obtained ASCs differentiated into osteogenic, chondrogenic, and adipogenic lineages, which was verified by means of specific staining (Figure 1(b)).

### 3.2. Proliferation Factor and miRNA Expression of ASC in Standard Culture

**Figure 2:**
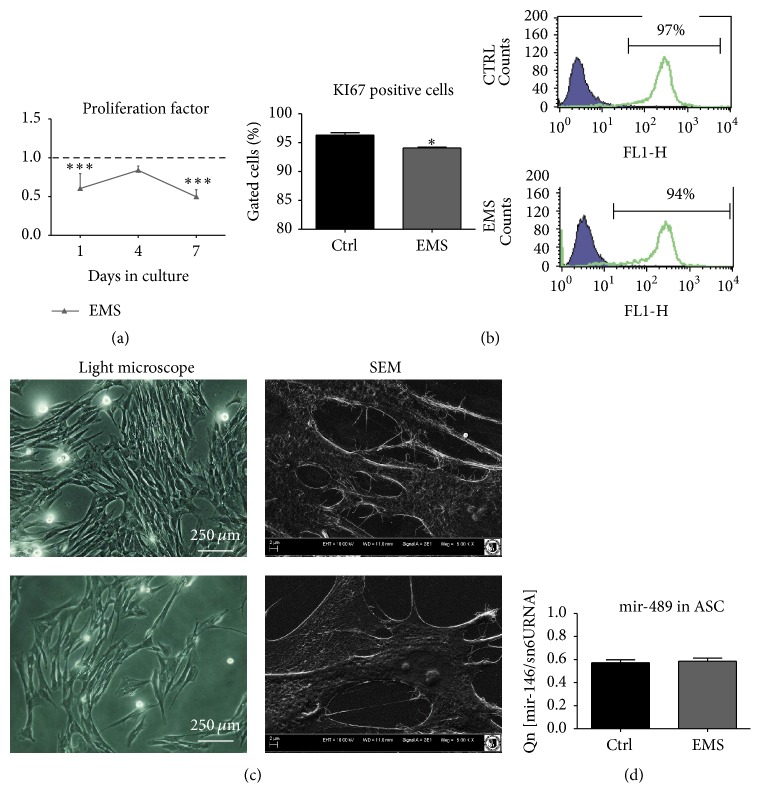
Proliferation and morphology of ASC. Using resazurin-based assay, the growth kinetics of isolated cells was established. During the seven-day test ASC_EMS_ displayed decreased proliferation potential in comparison to control group (a). Flow cytometry analysis confirmed decreased expression of Ki67 in those cells (b). Morphology of investigated cells was evaluated after day 7. While ASC_CTRL_ reached high confluence and developed robust net of cytoskeletal projections (lamellipodia and filopodia), ASC_EMS_ did not form multilayer and were more amorphous in shape with reduced cellular projections (c). Interestingly, there were no differences in the expression of mir-489, which plays crucial role in the differentiation process (d). Results are expressed as mean ± SD. ^*∗∗∗*^ *p* value < 0.001.

Viability characteristics of ASCs were assessed within seven days of culture. The number of viable cells in culture was evaluated with resazurin-based assay (TOX-8) in accordance to the manufacturer's protocol. Throughout the experiment, ASC_EMS_, displayed lowest proliferation rate in comparison to control group, and on the 1st and 7th days of the experiment, difference between groups was statistically significant (Figure 2(a), *p* < 0.001). Moreover, using flow cytometry, the expression of KI67 was estimated and confirmed the lower proliferation rate of ASC_EMS_ (Figure 2(b)). Cell morphology was evaluated on the last (7th) day of the experiment (Figure 2(c)). Observations with light microscope showed that ASC_CTRL_ displayed more fibroblast-like, elongated morphology, while ASC_EMS_ were flat and no longer bipolar with “fried-egg” like morphology. SEM analysis revealed that ASC_CTRL_ developed greatest net of cytoskeletal projections including lamellipodia and filopodia, which connected adjacent cells, in comparison to ASC_EMS_. Interestingly, we observed no differences in the expression of mir-489 in investigated cells (Figure 2(d)).

### 3.3. Evaluation of miRNA in Isolated MVs

Prior to the analysis of miRNA content, MVs were isolated form culture medium using DEX-PEG method as described above. We did not observe differences in mir-233 expression within investigated groups (Figure 2(a)). Interestingly, mir-489 was upregulated (*p* < 0.01, Figure 2(b)) while mir-146 was downregulated in MVs_EMS_ (*p* < 0.05, Figure 2(c)).

### 3.4. Mitochondria Condition and Clearance in ASC_CTRL_ and ASC_EMS_

**Figure 3:**
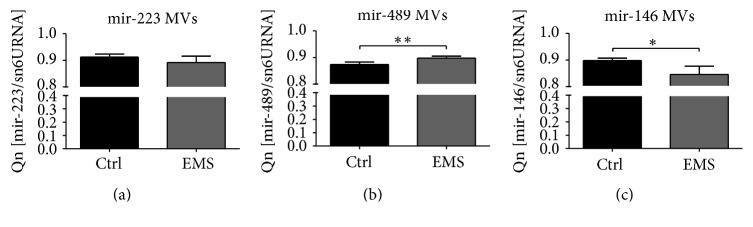
Evaluation of miRNA content in microvesicles (MVs) secreted by ASC_CTRL_ and ASC_EMS_. There were no significant differences in the expression of mir-223 in investigated groups (a). Interestingly, secreted in MVs, mir-489 was upregulated in ASC_EMS_ (b). On the contrary, mir-146, whose decreased expression is correlated with inflammation and diabetic wound healing, was downregulated in ASC_EMS_ (c). Results are expressed as mean ± SD. ^*∗*^ *p* < 0.05, ^*∗∗*^ *p* < 0.01.

**Figure 4:**
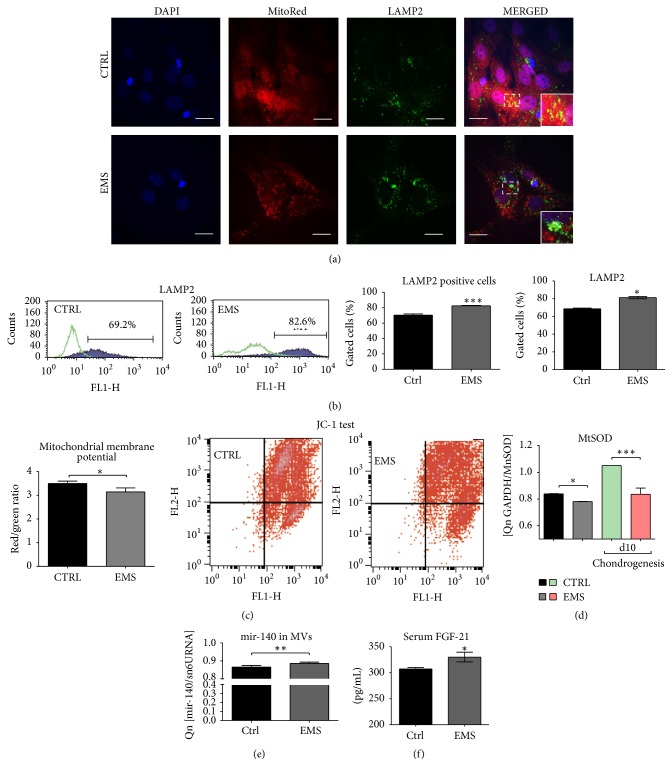
Mitochondria condition and clearance in ASC_CTRL_ and ASC_EMS_. Representative photographs showing the results of DAPI, MitoRed, and anti-LAMP2 stainings. Interestingly, although LAMP2 expression was increased in ASC_EMS_, clearance of mitochondria in those cells seems to be reduced as we observed decreased number of mitochondria fused with lysosomes in comparison to control group (a). Flow cytometry analysis confirmed increased expression of LAMP2 in ASC_EMS_ (b). Mitochondria deterioration was confirmed with flow cytometry using JC1 test, as we observed decreased mitochondrial membrane potential in ASC_EMS_ (c). Moreover, the antioxidative protection coming from mitochondrial MnSOD was reduced (d) as established with qRT-PCR. The mir-140 transported via MVs was increased in ASC_EMS_ (e) which correlates with increased expression of MFN. The serum level of FGF21, characteristic of obesity and diabetes, was upregulated in ASC_EMS_ (f). Results are expressed as mean ± SD. ^*∗*^ *p* < 0.05, ^*∗∗*^ *p* < 0.01, and ^*∗∗∗*^ *p* < 0.001. Scale bars: 10 *μ*m.

To evaluate the mitochondrial network and lysosome formation cells were stained with MitoRed and anti-LAMP2 antibody and visualized under confocal microscope. The mitochondria network in control group was more robust and the organelles were evenly dispersed throughout cell's body. In contrast, ASC_EMS_ were mainly observed around the nucleus area forming dense aggregates. The immunofluorescence stating for LAMP2 revealed increased lysosome formation in ASC_EMS_. Interestingly, merged photographs of MitoRed-LAMP2 showed that lysosomes containing mitochondria occurred more frequently in ASC_CTRL_ indicating on mitophagy deterioration in ASC_EMS_ (Figure 3(a)). Flow cytometry analysis confirmed increased expression of LAMP2 in ASC_EMS_ (Figure 3(b)). The JC-1 test confirmed mitochondria impairment in ASC_EMS_ (Figure 4(c)). Moreover, those cells were characterized by decreased expression of mitochondrial SOD (Figure 4(d)) during both control and chondrogenic conditions. Interestingly, MVs_EMS_ contained more mir-140 which is responsible for oxidative stress response in comparison to control group (Figure 4(e)). Serum FGF-21 which stands as one of the mitochondria impairment markers was increased in EMS horses serum (Figure 4(f)).

### 3.5. Endoplasmic Reticulum (ER) Stress and Autophagy

**Figure 5:**
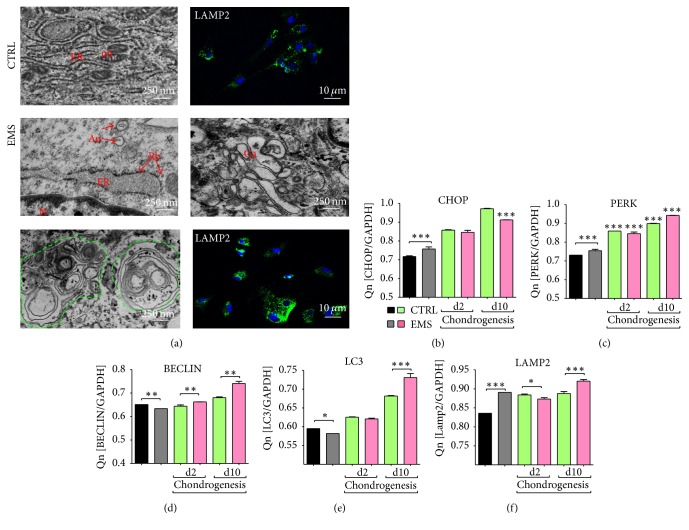
Endoplasmic reticulum (ER) stress and autophagy. The autophagy in investigated ASC was established using transmission and confocal microscopy (a). Both ER and Golgi compartments were grossly expanded in ASC_EMS_. Moreover, large part of ER become fragmented and disintegrated. Large double-membrane vacuoles, multilamellar autophagic bodies (green outline), preautophagosome structures, and engulfed organelles were characterized for ASC_EMS_. Moreover, anti-LAMP2 immunofluorescence staining revealed increased lysosome accumulation in those cells. The expression of ER stress-related genes—CHOP (b) and PERK (c)—was increased in ASC_EMS_ in control conditions but during chondrogenic differentiation the amount of its mRNA was decreased in comparison to control group. Transcription of genes involved in the autophagy process including Beclin (d) and LC3 (e) in standard culture was decreased in ASC_EMS_, but during chondrogenesis it significantly increased. mRNA level for LAMP2 was upregulated in those cells in both standard and chondrogenic cultures (f) despite day 2. Results are expressed as mean ± SD. ^*∗*^ *p* < 0.05, ^*∗∗*^ *p* < 0.01, and ^*∗∗∗*^ *p* < 0.001. Au: autophagosomes, Er: endoplasmic reticulum, Rb: ribosomes, N: nucleus, Ga: Golgi apparatus, Mt: mitochondria.

Using immunofluorescence staining for LAMP2 and TEM microscopy we evaluated the autophagy in investigated cells. Mitochondria in ASC_CTRL_ presented typical, bean-like shape with multiple cristae. ER was well developed with the presence of many ribosomes. On the contrary, in ASC_EMS_, ER and Golgi apparatus compartments were grossly expanded. Moreover, ER becomes fragmented and disintegrated. Large, double-membrane autophagic vacuoles and preautophagosome structures and multilamellar bodies were observed in those cells (Figure 5(a)). Moreover, staining for LAMP2 revealed increased lysosome formation in ASC_EMS_. Interestingly, qRT-PCR confirmed increased ER stress in ASC_EMS_ as we observed increased expression of CHOP (Figure 5(b)) and PERK (Figure 5(c)) mRNA during control and chondrogenic conditions. The expression of Beclin (Figure 5(d)) and LC3 (Figure 5(e)) was decreased in ASC_EMS_ in control culture but during chondrogenic conditions we observed upregulation of both transcripts. The mRNA levels of LAMP2 confirmed the immunofluorescence data as we observed increased expression of LAMP2 in ASC_EMS_ (Figure 5(f)).

### 3.6. Mitochondria Dynamics in ASC_CTRL_ and ASC_EMS_

**Figure 6:**
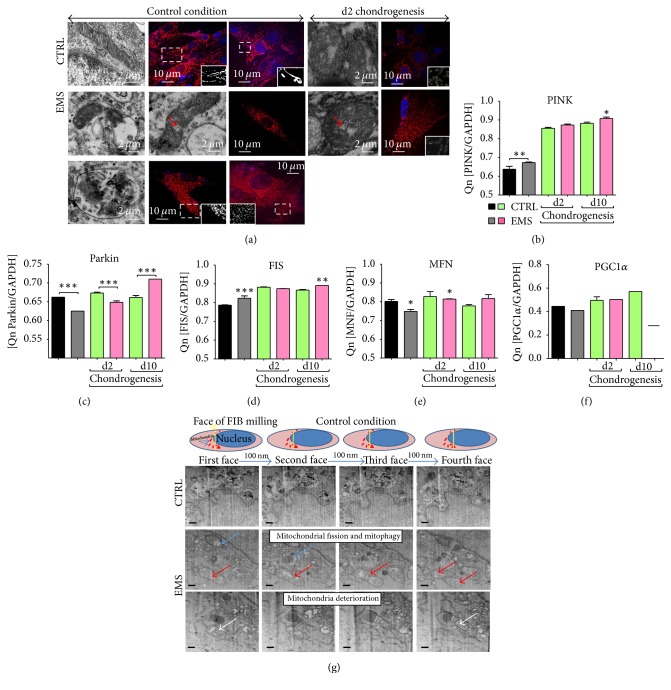
Mitochondria dynamics in ASC_CTRL_ and ASC_EMS_. Distribution of ASC mitochondria was evaluated using TEM and confocal microscopy with MitoRed staining (a). In control conditions ASC_CTRL_ mitochondria presented typical, elongated, bean shape morphology with well-developed cristae. A long net of connected mitochondria was observed although, during chondrogenesis, fragmented mitochondrial phenotype was observed. In ASC_EMS_, mitochondrial fission was enhanced during both control and chondrogenic conditions. Using RT-PCR we evaluated the expression of PINK (b), PARKIN (c), FIS (d), MFN (e), and PGC1*α* (f) during control and chondrogenic conditions. Using SEM-FIB we evaluated ultrastructure of investigated cells (g). ASC_EMS_ were characterized by increased fission (red arrow), mitophagy (blue arrow), and fragmented and deteriorated mitochondrial phenotype with few cristae (white arrow). Scale bars: 250 nm. ^*∗*^ *p* < 0.05, ^*∗∗*^ *p* < 0.01, and ^*∗∗∗*^ *p* < 0.001.

Using confocal and TEM microscopy we evaluated mitochondria dynamics and network in investigated cells. In control conditions, ASC_CTRL_ mitochondria presented typical bean-like elongated morphology, forming the long tubular network. On the contrary, ASC_EMS_ mitochondria were characterized by membrane raptures and vacuole formation. Many of them were packed into autophagolysosomes. The mitochondrial net was fragmented, suggesting increased mitochondrial fission (Figure 6(a)). Interestingly, during chondrogenesis, we observed fragmentation of mitochondrial network in both of the investigated groups. Moreover, using qRT-PCR, we estimated the expression of genes involved in mitochondrial biogenesis and dynamics. Interestingly, the expression of PINK (Figure 6(b)) was increased, but Parkin (Figure 6(c)) decreased in ASC_EMS_. Decreased expression of Parkin may be partially responsible for accumulation of impaired mitochondria in ASC_EMS_. To confirm the data from confocal microscope, we investigated the expression of FIS and MNF mRNA to provide more quantitative data about mitochondria dynamics. The mRNA level of FIS was increased in ASC_EMS_ (Figure 6(d)) which confirmed increased mitochondria fission in those cells. During chondrogenesis its expression was only statistically significant on day 10. On the contrary, the expression of MNF, related to the mitochondrial fusion, was increased in ASC_CTRL_ (Figure 6(e)). No differences were observed in PGC1*α* expression (Figure 6(f)). Moreover, using FIB-SEM technique, formation of mitophagosomes and clearance of deteriorated mitochondria in ASC_EMS_ were assessed (Figure 6(g)).

### 3.7. Chondrogenic Differentiation of ASC_CTRL_ and ASC_EMS_

**Figure 7:**
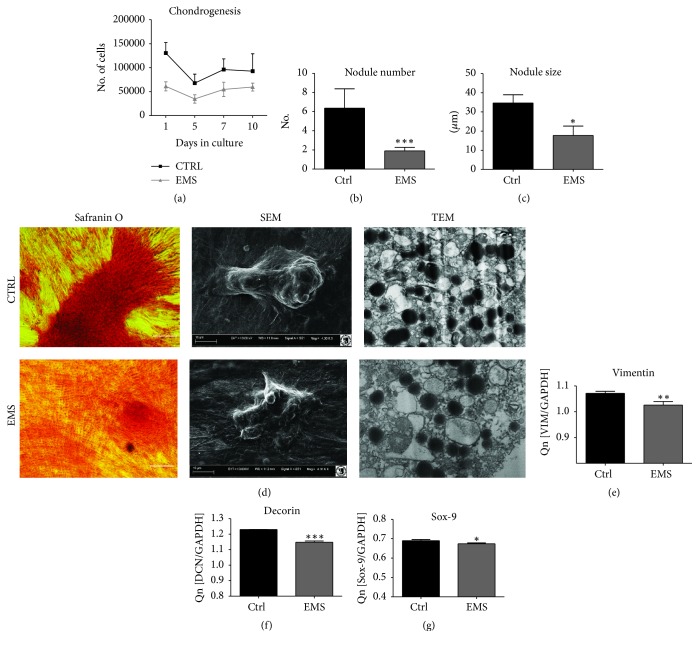
Chondrogenic differentiation of ASC_CTRL_ and ASC_EMS_. During chondrogenesis ASC_EMS_ displayed decreased proliferation potential in comparison to control group (a). The number (b) and size (c) of cartilaginous nodule were reduced in that group. Visualization of cartilaginous nodules with Safranin O and SEM confirmed that ASC_CTRL_ underwent more effective chondrogenesis in comparison to control group. Formation of proteoglycan-rich extracellular matrix was also confirmed using TEM (d). The expression of chondrogenic markers including Vimentin (e), Decorin (f), and Sox-9 (g) was decreased in ASC_EMS_, impinging on chondrogenesis impairment and multipotency deterioration. Results are expressed as mean ± SD. ^*∗*^ *p* < 0.05, ^*∗∗*^ *p* < 0.01, and ^*∗∗∗*^ *p* < 0.001.

ASC were cultured under chondrogenic conditions in order to evaluate their chondrogenic differentiation potential on a functional level. A significantly lower proliferative activity was observed in chondrocytes precursors (Chp) derived from ASC_EMS_ throughout the whole experiment (Figure 7(a), *p* < 0.001). Additionally using SEM we evaluated the number and size of cartilaginous nodules within investigated cells. Hence we observed that ASC_EMS_ were characterized by the formation of lowest number of nodules (Figure 7(b), *p* < 0.001). Moreover, those nodules presented decreased size in comparison to ASC_CTRL_ (Figure 7(c)). Stainings with Safranin O confirmed that formation of proteoglycan-enriched matrix was more robust in ASC_CTRL_. Similarly, SEM and TEM imaging confirmed that effectiveness of differentiation process was greater in ASC_CTRL_ (Figure 7(d)). To provide quantitative data, qRT-PCR was performed. The expression of Vimentin (Figure 7(e), *p* < 0.01), Decorin (Figure 7(f), *p* < 0.001), and Sox-9 (Figure 7(f), *p* < 0.05) was significantly reduced in ASC_EMS_.

### 3.8. miRNA Expression during Chondrogenic Differentiation

**Figure 8:**
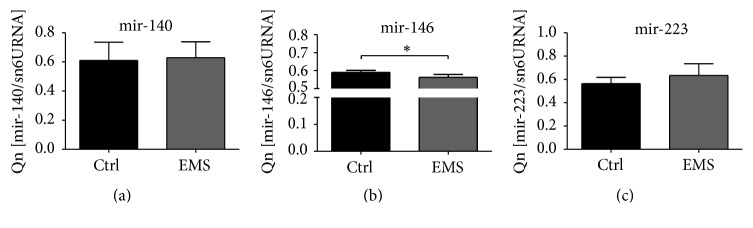
miRNA expression during chondrogenic differentiation. Using qRT-PCR miRNA in chondrocytes precursors was established after day 10. No differences in mir-140 (a) and mir-223 were observed (c). Only mir-146 expression was significantly reduced in ASC_EMS_ group (b). Results are expressed as mean ± SD. ^*∗*^ *p* < 0.05.

On the last day of chondrogenic differentiation, we estimated the miRNA expression in investigated cultures. Interestingly, no differences were observed in the expression of mir-140 (Figure 8(a)) and mir-223 (Figure 8(c)). Only mir-146 expression showed statistical significance as it was decreased in ASC_EMS_ (Figure 8(b), *p* < 0.05).

### 3.9. Analysis of the SOD, ROS, and NO in Chondrocytes Precursors

**Figure 9:**
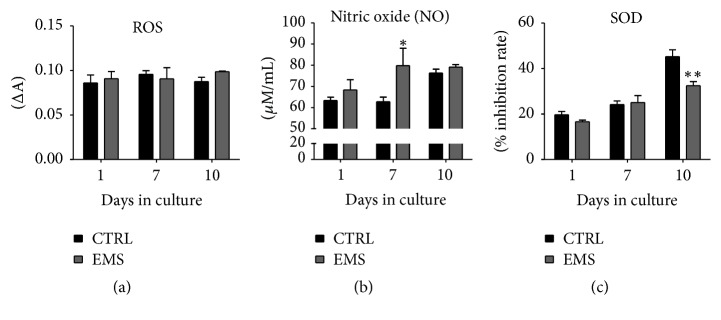
Analysis of the ROS, NO, and SOD activity in chondrocytes precursors. The ROS levels during chondrogenesis were similar in both investigated groups (a). Nitric oxide (NO) concentration was increased in ASC_EMS_, but only at day 7 it was statistically important. Antioxidative protection coming from SOD was also reduced in those cells, especially after day 10. Results are expressed as mean ± SD. ^*∗*^ *p* < 0.05, ^*∗∗*^ *p* < 0.01.

Oxidative stress factors, for example, ROS and NO, and the activity of SOD were evaluated after the 1st, 7th, and 10th days of culture. Obtained results shows that, during osteogenesis, the amount of ROS is similar between groups (Figure 9(a)). Interestingly, in the course of osteogenic differentiation process, the amount of NO (Figure 9(b)) and SOD (Figure 9(c)) increases. The NO levels were significantly increased in ASC_EMS_ after the 7th day (*p* < 0.05), while SOD activity was significantly decreased after the 10th day (*p* < 0.01).

### 3.10. The Expression of HIF-1-*α* and FOXO1

**Figure 10:**
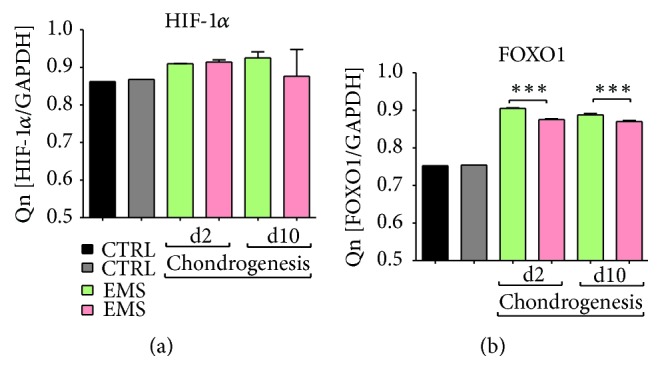
The expression of HIF-1-*α* and FOXO1. No differences in the mRNA levels of HIF-1-*α* were observed among investigated groups during both control and chondrogenic conditions (a). FOXO1 expression was downregulated in ASC_EMS_ after 2nd and 10th days of chondrogenesis (b). Results are expressed as mean ± SD. ^*∗∗∗*^ *p* < 0.001.

Using RT-PCR the mean mRNA levels of HIF-1-*α* (Figure 10(a)) and FOXO1 (Figure 10(b)) were established. We observed no differences in the expression of HIF-1-*α* in control and chondrogenic conditions between groups. The expression of FOXO1 in control condition was comparable between groups. During chondrogenesis it was significantly decreased in ASC_EMS_ (*p* < 0.001) after the 2nd and 10th days, respectively.

## 4. Discussion

Mesenchymal stem cells harvested from adipose tissue (ASCs) contribute to peripheral tissue repair* in vivo* and become a cell source for regenerative medicine, that underlines their importance in the field of veterinary regenerative medicine [2, 22]. However, EMS as a serious endocrine disorder, which is steadily growing, has unquestioned impact on ASCs cytophysiology [10]. As it was previously demonstrated ASC of EMS horses (ASC_EMS_) are marked by excessive molecular aging, decreased viability, displayed senescence associated features, and abundantly accumulated oxidative stress factors [10]. Hence, we were interested whether ASC_EMS_ are characterized by impaired chondrogenic differentiation potential, since those cells are commonly used for the regenerative medicine purposes. Here we demonstrated that ASC_EMS_ displayed slightly impaired chondrogenic differentiation potential, but abundance of auto- and mitophagy are the processes that allowed ASC_EMS_ to maintain multipotency abilities. Selective autophagy in the course of equine metabolic syndrome might become one of the fundamental mechanisms that protects progenitor cells of adipose tissue from cellular senescence and cytophysiological impairment.

Disease and aging are two major factors that have profound influence on adipose tissue progenitor cells characteristics. Here we have found that ASC_EMS_ in native culture are characterized by decreased viability and clonogenic potential as well as exhibit lower percentage of Ki67 positive cells when compared to the healthy ASC. Moreover, among typical for mesenchymal stem cells surface antigens (i.e., CD44+, CD105+, and CD90+) we have found the significant higher fraction of CD44+ cells among investigated ASC_EMS_ population. It was recently demonstrated that CD44+ is overexpressed in inflammatory cells in obese patients adipose tissue and serum CD44+ cells level is positively correlated with insulin resistance and glycemic control [23]. Additionally, it was shown that CD44+ likely plays a causative role in the development of adipose tissue inflammation and insulin resistance in rodents and humans [24]. Thus, obtained data confirms the above-mentioned results and indicates that CD44+ might be useful marker for ASC_EMS_ characterization. Simultaneously, with decreased ASC_EMS_ proliferative potential, we have noticed their impaired growth pattern and limited extracellular microvesicles (MVs) secretion. Moreover, the MVs derived from ASC_EMS_ were characterized by the decreased amount of transferred mir-223 and mir-146 with simultaneous upregulation of mir-456 mRNA level. This stands in good agreement with other authors' previous finding, since mir-223 and mir-146 were found to be characteristic for obese, hypercholesterolemic individuals [25, 26]. What is more, as mentioned above mir plays crucial role in switching on proinflammatory genes expression. It stands in good agreement and fulfilled our previous findings, where we showed that native ASC_EMS_ were characterized by upregulation of p21, p53, and cas-9 as well as BAX expression in comparison to healthy ASC [10].

The impaired proliferative activity, cell growth pattern, and apoptosis observed in ASC_EMS_ might be directly linked to oxidative stress factors (OS) accumulation. However, one of the mechanisms that protects progenitor cells against excessive accumulation of OS factors and its harmful effects is called mitophagy. In the current research, we observed the reduced number of mitochondria with parallel higher expression of lysosomal associated membrane protein 2 (LAMP2) in ASC_EMS_. Observed mechanism impinges on “autophagic flux” in ASC_EMS_ when compared to the control cells. Moreover, we observed significantly decreased mitochondrial membrane potential (JC-1 test), which may be partially responsible for entering of ASC_EMS_ in autophagic pathway due to mitochondria impairment. Interestingly, ASC_EMS_ displayed lower expression of MnSOD, which can lead to decreased antioxidative protection of mitochondria and in consequence switch autophagy in those cells on. In native, nonchondrogenic culture in both Beclin1 and LC3 was downregulated in ASC_EMS_ that partially excludes autophagy and indicates regular lysosomic fusion. Moreover, observed upregulation of mir-140, which exerts effect on mitochondrial fission and apoptosis through targeting mitofusin 1 (Mfn1), may be directly correlated with frequently observed in ASC_EMS_ mitochondrial fission. What is interesting, we simultaneously observed increased amount of serum fibroblast growth factor 21 (FGF21) in EMS horses. It was shown that FGF21 is a hormone that mediates an adaptive response to starvation and becomes also a long-standing marker of mitochondrial disease in humans [27, 28]. The elevated levels of FGF21 were observed in people suffering from mitochondrial diseases standing as a novel biomarker of mitochondrial disorders. On the contrary, some studies showed that FGF21 is a promising dietary restriction mimetic that can increase life span and health span benefits [29]. Because of dual role of FGF21 and the contractionary data it is difficult to establish its role in EMS horses, although data presented by Wall et al. [30] showed FGF21 longevity effects as it helped to maintain mitochondria metabolic homeostasis in polymerase gamma mtDNA mutator (POLG) mice. Interestingly, the hormone was highly active despite the mice accelerated aging. It is tempting to speculate that similar, paradox, mechanism is switched on in EMS horses, standing as an adaptation to help animals maintain their metabolic help. Moreover, feeding mice on high-fat diet accelerated FGF21 beneficial effect; thus activating FGF21 by deteriorated mitochondria may be indeed the protective mechanism in individuals suffering from diabetes and metabolic syndrome, especially the fact that mitochondrial dysfunction in both of these diseases was proved in many studies [10, 31–33].

However, lysosomal degradation of impaired mitochondria, as a process of damaged mitochondria elimination, seems to be characteristic for native ASC_EMS_ culture. The situation is changed, when chondrogenic differentiation potential is considered.

Besides its recycling functions, in response to energy or nutrients deficiency, autophagy is also recognized as a quality control mechanism for proteins and organelles including mitochondria [34–36]. It may be induced as a result of cellular stress such as ROS accumulation and ER stress, which correlates with our data, as we observed increased expression of autophagy-essential genes in ASC_EMS_. Activation of autophagy in those cells may help to clear damage due to ER stress proteins and impaired by ROS mitochondria. Therefore, autophagy is crucial for ASC_EMS_ to maintain their “stemness,” although the role of that process in regulation of stem cells metabolism is still poorly understood. Available data suggest that unique properties of stem cells including pluripotency, self-renewal, and differentiation depend on autophagy activation [37]; thus we evaluated how the expression of autophagy-essential genes changes during chondrogenesis. Another recent report showed that autophagy protects rat bone marrow derived MSC from both hypoxia and serum deprivation [38]. Thus, it is enticing to speculate that autophagy is also protective mechanism that allows ASC_EMS_ to fulfil their function in the unfavorable environment of adipose tissue inflammation. Autophagy was also shown to participate in differentiation, as a cell remodeling mechanism that promotes morphological and structural changes. Here we have found that during chondrogenesis expression of Beclin1 and LC3 increases which stands to the contrary of the data presented by Oliver et al. [39], who observed that autophagy decreases during differentiation of MSC isolated from humans. Those discrepancies may result from distinct time points for each experiment performed. It was shown that that during terminal differentiation of reticulocytes into erythrocytes, mitochondria are eliminated in an autophagy-dependent fashion [40]. Here we have found that ASC_EMS_ characterized by mitochondria impairment and ROS accumulation undergo mitochondria clearance via mitophagy as observed with TEM, during both control and chondrogenic conditions. Thus, considering that some amino acids generated through the autophagic pathway may be used for protein, nucleotides, sterols, and energy synthesis, it is tempting to speculate that autophagy may help ASC_EMS_ undergo differentiation. The “autophagic switch” may lead to synthesis of proteins and other cellular constituents, in addition to degrading proteins related to pluripotency maintenance that may impair differentiation. It has been shown that adipose-specific deletion of Atg7 leads to decreased adipose mass and enhanced insulin sensitivity, and autophagy is important in normal adipogenesis [41]. On the one hand, silencing of Beclin1 results in enhanced chondrocyte death [42]. Increased in ASC_EMS_ autophagy may result from ER stress at it was shown that autophagy is activated for cell survival during such condition [43]. Recently, Kouroku et al. reported that expanded polyglutamine- (polyQ-) induced ER stress activates autophagosome formation with LC3 conversion from LC3-I to -II via the PERK-eIF2*α* pathway [44]. It stands with good agreement with our data as we observed upregulation of both PERK and CHOP mRNA in ASC_EMS_. Despite being merely speculation, autophagy could help ASC_EMS_ maintain their self-renewal and pluripotency capacity or also differentiate by providing ATP and precursors for protein synthesis.

Mitochondrial dynamics was recently recognized as an important constituent of cellular quality control [45]; thus here, for the first time, we evaluated mitochondria dynamics and localization in ASC during control and chondrogenic conditions. Accumulating body of evidence impinges on vital role of mitochondria in metabolic homeostasis maintenance and differentiation. Mitophagy has been implicated in regulation of mitochondria number and distribution during hematopoietic stem cells differentiation [46]. Here we have found that, during chondrogenesis, mitochondria undergo more frequent fission in both of investigated ASC groups. Interestingly, in control condition, increased fission was observed in ASC_EMS_ which may be correlated with apoptosis and decreased proliferation of those cells. Our data stands with a good agreement with Forni et al. [47] who observed that mitochondria were more rounded and fragmented during commitment toward chondrogenesis although in murine dermal MSC differentiation. The remodeling of the mitochondrial network is essential for the differentiation program, since these cells' capacity to differentiate when fusion or fission is abrogated. Moreover, activation of mitophagy and inhibition of mitochondria biogenesis may reflect physiologically programmed step toward maturation under hypoxia which occurs* in vivo *[48]. However, a deep relationship between mitochondria dynamics and network in ASC during health and disease or differentiation needs to be further elucidated.

The past decade has witnessed significant growth in interest regarding stem cells and autophagy. However, research of those two exciting areas is very much in its infancy. Here, for the first time, we evaluated autophagy, ER stress, and mitochondria dynamics in ASC during both control and chondrogenic conditions. During chondrogenesis we observed “autophagic switch” in both healthy and EMS groups, while basal autophagy was increased in ASC_EMS_. As stem cells destined for regenerative medicine applications may be generated from individuals of advanced age or suffering from metabolic disorders (autophagic/mitophagic), quality control processes would need to be carefully examined and controlled to ensure long-term safety of any cellular grafts. It is tempting to speculate that modulation of autophagy may have important outcomes, such as an increase in the efficiency of cell proliferation and differentiation.

## Figures and Tables

**Table 1:** Criteria for dividing the horses into the experimental and control groups.

Group	O (number)	Sex	Main clinical parameters					
			Bw (Kg)	BCS (1–9)	CNS (1–5)	Fasting insulin (mU/mL)	LEP (ng/mL)	CGIT : GLU in 45 min (mg/dL)
Healthy horse	1	f	610	6	1	7	3.21	74/p
	2	f	644	7	2	12	4.12	69/p
	3	f	627	7	2	9	2.87	71/p
	4	m	609	6	1	8	1.86	89/p
	5	m	649	7	2	14	3.56	80/p
	6	m	639	6	2	13	2.91	74/p

Mean ± SD			629.7 ± 15.7	6.5 ± 0.5	1.7 ± 0.5	10.5 ± 2.6	3.1 ± 0.7	76.2 ± 6.7

Horse with EMS	1	f	710	8	3	83	4.89	138/p
	2	f	726	9	3	67	5.19	141/p
	3	f	760	9	4	98	9.12	140/p
	4	m	709	8	3	73	8.49	136/p
	5	m	716	8	4	69	7.27	134/p
	6	m	746	9	4	82	8.36	146/p

Mean ± SD			727.8 ± 19.1	8.5 ± 0.5	3.5 ± 0.5	78.7 ± 10.5	7.2 ± 1.6	139.2 ± 3.8

f: female, m: male, BW: body weight, BCS: body condition score, CNS: cresty neck score, CGIT: combined glucose-insulin test, SD: standard deviation, LEP: leptin, GLU: glucose, p: positive test results, and n: negative test results.

**Table 2:** Sequences of primers used in qPCR.

Gene	Primer	Sequence 5′-3′	Amplicon length (bp)	Accession number
MnSOD	F	CAATAAGGACCAGGGACGCC	244	XM_014858346.1
	R	GCTTAATGCACTCGGTGTAACG		
Parkin	F	TCCCAGTGGAGGTCGATTCT	218	XM_005608126.2
	R	CCCTCCAGGTGTGTTCGTTT		
PGC1*α*	F	TCTACCTAGGATGCATGG	93	XM_005608845.2
	R	GTGCAAGTAGAAACACTGC		
HIF-1-*α*	F	CTCAAATGCAAGAACCTGCTC	86	XM_014735822.1
	R	TTCCATACCATCTTTTGTCACTG		
Vimentin	F	GCAGGATTTCTCTGCCTCTT	352	XM_014852743.1
	R	TATTGCTGCACCAAGTGTGT		
FOXO1	F	ATTGAGCGCTTGGACTGTGA	311	XM_014732057.1
	R	CGCTGCCAAGTTTGACGAAA		
LC3	F	TTACTGCTTTGCTCTGCCAC	213	XM_005608485.2
	R	AGCTGCTTCTCCCCCTTGT		
Beclin	F	GATGCGTTATGCCCAGATGC	147	XM_014729146.1
	R	ATCCAGCGAACACTCTTGGG		
LAMP2	F	GCACCCCTGGGAAGTTCTTA	139	XM_014733098.1
	R	TTCGAGGATCTGTGCCAATCA		
GAPDH	F	GATGCCCCAATGTTTGTGA	250	NM_001163856.1
	R	AAGCAGGGATGATGTTCTGG		
CHOP	F	AGCCAAAATCAGAGCCGGAA	272	XM_014844003.1
	R	GGGGTCAAGAGTGGTGAAGG		
PERK	F	GTGACTGCAATGGACCAGGA	283	XM_014852775.1
	R	TCACGTGCTCACGAGGATATT		
PINK	F	GCACAATGAGCCAGGAGCTA	298	XM_014737247.1
	R	GGGGTATTCACGCGAAGGTA		
PARKIN	F	TCCCAGTGGAGGTCGATTCT	218	XM_014858374.1
	R	CCCTCCAGGTGTGTTCGTTT		
FIS	F	GGTGCGAAGCAAGTACAACG	118	XM_001504462.4
	R	GTTGCCCACAGCCAGATAGA		
MFN	F	AAGTGGCATTTTTCGGCAGG	217	XM_001495170.5
	R	TCCATATGAAGGGCATGGGC		
Decorin	F	GATGCAGCTAGCCTGAGAGG	248	XM_014841263.1
	R	GTGTTGTATCCAGGTGGGCA		
Sox-9	F	GAACGCCTTCATGGTGTGGG	225	XM_014736619.1
	R	TTCTTCACCGACTTCCTCCG		

Sequences, amplicon length, and accession numbers of the primer sets. MnSOD: mitochondrial superoxide dismutase 2; Parkin: parkin RBR E3 ubiquitin protein ligase (PARK2); PGC1 *α*: peroxisome proliferator-activated receptor gamma, coactivator 1 alpha; HIF-1-*α*: hypoxia inducible factor 1, alpha subunit; Bcl-2: B-cell CLL/lymphoma 2; FOXO1: forkhead box O1; LC3: microtubule associated protein 1 light chain 3 beta (MAP1LC3B); Beclin: beclin 1, autophagy related (BECN1); LAMP2: lysosomal-associated membrane protein 2; GADPH: glyceraldehyde-3-phosphate dehydrogenase; CHOP: DNA damage inducible transcript 3 (DDIT3); PERK: eukaryotic translation initiation factor 2-alpha kinase 3; PINK: PTEN-induced putative kinase 1; PARKIN: parkin ligase; FIS: fission, mitochondrial 1; MFN: mitofusin 1 (MFN1); Sox-9: transcription factor SOX-9.

**Table 3:** Sequences of primers used in miRNA expression analysis.

Primer miRNAs	Sequence 5′-3′	Accession number
miR-140-3p	TACCACAGGGTAGAACCACGGA	MI0012682
miR-146a-5p	TGAGAACTGAATTCCATGGGTT	MI0012809
miR-223-3p	TGTCAGTTTGTCAAATACCCCA	MI0012953
miR-489-3p	GTGACATCACATATACGGCAGC	MIMAT0012943
