# Corrigendum to “Macroautophagy and Selective Mitophagy Ameliorate Chondrogenic Differentiation Potential in Adipose Stem Cells of Equine Metabolic Syndrome: New Findings in the Field of Progenitor Cells Differentiation”

**DOI:** 10.1155/2017/3861790

**Published:** 2017-07-27

**Authors:** Krzysztof Marycz, Katarzyna Kornicka, Jakub Grzesiak, Agnieszka Śmieszek, Jolanta Szłapka

**Affiliations:** ^1^Electron Microscopy Laboratory, The Faculty of Biology and Animal Science, Wroclaw University of Environmental and Life Sciences, Wroclaw, Poland; ^2^Wroclaw Research Centre EIT+, Wroclaw, Poland

In the article titled “Macroautophagy and Selective Mitophagy Ameliorate Chondrogenic Differentiation Potential in Adipose Stem Cells of Equine Metabolic Syndrome: New Findings in the Field of Progenitor Cells Differentiation” [1], an acknowledgment should be added as follows:

## References

[1] Marycz K., Kornicka K., Grzesiak J., Śmieszek A., Szłapka J. (2016). Macroautophagy and selective mitophagy ameliorate chondrogenic differentiation potential in adipose stem cells of equine metabolic syndrome: new findings in the field of progenitor cells differentiation. *Oxidative Medicine and Cellular Longevity*.

